# Algorithmic approach to find S-consistency in Common-Edge signed graph

**DOI:** 10.1016/j.mex.2022.101783

**Published:** 2022-07-21

**Authors:** Anshu Sethi, Deepa Sinha, Obaidullah Wardak

**Affiliations:** a The North Cap University, India; b South Asian University, New Delhi, India

**Keywords:** Algorithm, Signed graph, Common-Edge signed graph, S-consistent, Consistent marked graph, Negative section

## Abstract

Common-Edge signed graph $C_{E}\left(S\right)$ of a signed graph S is a signed graph whose vertex-set is the pairs of adjacent edges in S and two vertices are adjacent if the corresponding pairs of adjacent edges of S have exactly one edge in common, with the sign same as that of Common-Edge. S-*Marked* signed graph T is a signed graph which receives the marking μ due to the signed graph S called marker. Further, T is S-*consistent* if a marker S is defined and if S-*marking*μof T with respect to which marked signed graph $T_{\mu }$ is consistent. In this paper, we give an algorithm to detect if $C_{E}\left(S\right)$ is S-consistent or not and determine its complexity.

• Algorithm to detect if $C_{E}\left(S\right)$ is S-consistent or not.

• Determination of algorithm's complexity.

## Graphical abstract

**Figure:**
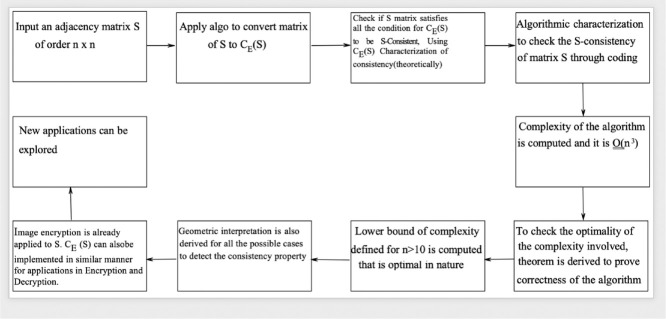

Specifications table

**Table:** 

Subject Area:	*Mathematics*
More specific subject area:	*Graph Theory*
Method name:	*Graph Algorithm*
Name and reference of original method:	*N/A*
Resource availability:	*Not applicabl*

## Introduction

We use definitions and notations of graph theory from the books of Harary [6] and West [15]. For the algorithms, Coreman [3] and Golumbic [5] are followed. A signed graph [16,17] is an ordered pair $S=(S^{u},\;\sigma )$, where $S^{u}=\left(V,E\right)$ is the underlying graph of S and σ:E→{+,−} is a function from edge set E of $S^{u}$ into the set {+,−}, called the sign function of S.

**Fig. 1:**
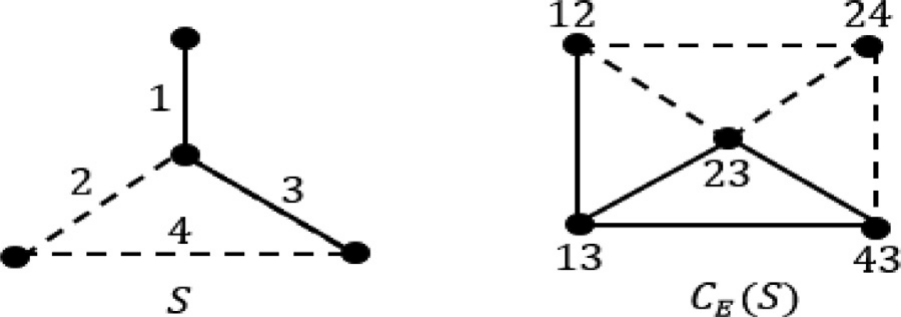
Common-Edge signed graph $C_{E}\left(S\right)$ of a given signed graph S.

Common-Edge signed graph [2], $C_{E}\left(S\right)$ of a signed graph S is a signed graph where vertex-set is the set of pairs of adjacent edges in S and two vertices are adjacent if the corresponding pairs of adjacent edges of S have exactly one edge in common, with the same sign as that of Common-Edge (see Fig. 1).

A marked signed graph [1] is an ordered pair Sμ=(S,μ) where $S=(S^{u},\;\sigma )$ is a signed graph and μ:V(S)→{+,−} is a function from the vertex set V(S) of S into the set {+,−}, called a marking of S. It can also be defined as a signed graph, each vertex of which is marked+ or −, and it is consistent if every cycle in the signed graph possesses an even number of ′−′ marked vertices. T is S-marked [1] if the vertices of T are marked due to the specific rule on signs of the edges of some signed graph S, called marker of T. Further, T is S-consistent whenever a marker S is to be specified, if there is an S-marking μ of T with respect to which the marked signed graph $T_{\mu }$ is consistent. Further, for a given signed graph S we define a function μ as:

$$\mu :V\left(C_{E}\left(S\right)\right)\to \left\{+,-\right\}$$

Such that for every $v\left(e_{i}e_{j};\;e_{i},\;e_{j}\in E\left(S\right)\right)\in V\left(C_{E}\left(S\right)\right)$,

$$\mu \left(u\right)=\left\{ \;\;\;\begin{array}{cc} - & if\;\;\sigma \left(e_{i}\right)=\sigma \left(e_{j}\right)=-\\ + & otherwise. \end{array}\right.$$

And look for the algorithm so that the resulting marked signed graph ${\left(C_{E}\left(S\right)\right)}_{\mu }$ is S-consistent.

Gill & Patwardhan [4] defines *negative section* of a subsigned graph S′ of a signed graph S as a maximal connected edge-induced subsigned graph in S′ consisting of only the negative edges ofS. A negative section in a cycle of S is essentially a maximal all-negative path in the cycle or the whole cycle itself. A cycle is positive if and only if it has an even number of negative sections of odd length.

Humans have cognitions which they invoke to develop attitudes towards various entities, including persons, in their perceivable environment. This is the foundation of consistency theory in social psychology that begins with the postulation that, over reasonably long periods of interaction with such entities, these attitudes might become so much a part of the identifiable characteristics of the individuals that they might be broadly categorized as being positive or negative in their overall attitudinal dispositions. Accordingly, each individual in a social group may be attributed a label ‘+' or ‘−', generically called a *mark*, according to whether the individual's such overall attitude is positive or negative. When such attitude gets expressed by an individual A in respect of another individual(or entity in the individual's immediate environment) B, we talk of ’attitudinal disposition of A towards B with its nature believed to be in consonance with A’s attitude; this real-life situation is fully representable as an arc (A,B) each of whose elements A, B and the arc (A,B) carries one of the values (or, ‘signs’) in the set M={+,−} representing the qualitative nature of attitudes possessed by the entities or expressed by them.

The concept of consistency is a motivation of communication networks. If binary messages with vertices having negative marking are sent through a network, reversing messages and vertices having positive marking, leaving them unchanged, then a consistent marked graph follows the consistency property: If a message is sent from the vertex u to the vertex v through two different vertex disjoint paths and u and v have the same sign, then v will receive the same message no matter which path is followed. In a similar manner, consistent marked graphs have utility in social networks, networks whose vertices are people. If some people always lie and some always tell the truth, a consistent social network has the property that if a message is sent from u to v and they have the same sign, then v will receive the same message independent of the path followed.

The S-consistent property is well defined in [2,9] which has a reference to [1] in which consistent marked graphs are considered. The property can be used in communication networks. Let us take an example of a network. The network can be represented by S. Encryption and decryption of S can be done to $\left(C_{E}\left(S\right)\right)$ and vice versa. The additional use of consistency property on S makes the network more secured. An optimal algorithm is developed with complexity $O(n^{3})$ for security of network and a new technique is defined to signed graphs in encryption and decryption process.

Thus, we define algorithms for network as Common-Edge signed graph with the additional property of S-consistency to social networks.

In this paper, an algorithm is developed using the characterization given by [1,2] to check if given Common-Edge signed graph is S-consistent or not. The paper was presented in the International Conference [14] and was highly appreciated. An algorithm to convert S to $C_{E}(S$) has already been discussed in [11] where the complexity involved in the algorithm is $O(n^{3})$.

## Characterization of S-consistent Common-Edge signed graphs

Theorem 2.1 *Acharya and Sinha* *[2]* *For any signed graph* S *,* $C_{E}\left(S\right)$ *is* S *-consistent if and only if the following conditions holds on* S *,*

(a) for each cycle Z in S,
  (i) if Z is all-negative, then it must be of even length;
  (ii) if Z is heterogeneous then the number of negative sections of length > 1 in Z and the total number of negative edges in the negative sections of length > 1 in Z and are of the same parity;
(b) for every vertex v∈V(S) with d(v)≥3,
  (i) $d^{-}\left(v\right)\leq 1$;
  (ii) size of any negative section containing v is at most one.

### Numerical interpretation

**Fig. 2:**
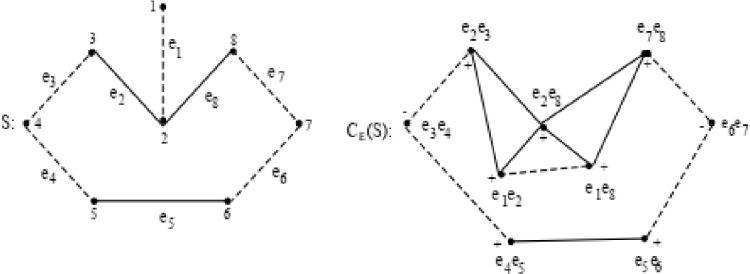
Signed graph S and $C_{E}\left(S\right).$

Consider the following signed graph S and $C_{E}\left(S\right)$ as shown in Fig. 2.

**Fig. 3:**
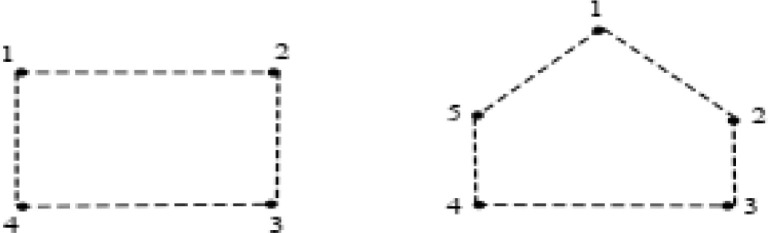
Example 1.

Here S has the adjacency matrix:

$$S=\left[\begin{array}{cccccccc} 0 & -1 & 0 & 0 & 0 & 0 & 0 & 0\\ -1 & 0 & 1 & 0 & 0 & 0 & 0 & 1\\ 0 & 1 & 0 & -1 & 0 & 0 & 0 & 0\\ 0 & 0 & -1 & 0 & -1 & 0 & 0 & 0\\ 0 & 0 & 0 & -1 & 0 & 1 & 0 & 0\\ 0 & 0 & 0 & 0 & 1 & 0 & -1 & 0\\ 0 & 0 & 0 & 0 & 0 & -1 & 0 & -1\\ 0 & 1 & 0 & 0 & 0 & 0 & -1 & 0 \end{array}\right]$$

The adjacency matrix corresponding to Common-Edge signed graph $C_{E}\left(S\right)$ is:

$$C_{E}\left(S\right)=\left[\begin{array}{ccccccccc} 0 & -1 & 1 & 1 & 0 & 0 & 0 & 0 & 0\\ -1 & 0 & 0 & 1 & 1 & 0 & 0 & 0 & 0\\ 1 & 0 & 0 & 1 & 0 & -1 & 0 & 0 & 0\\ 1 & 1 & 1 & 0 & 1 & 0 & 0 & 0 & 0\\ 0 & 1 & 0 & 1 & 0 & 0 & 0 & 0 & -1\\ 0 & 0 & -1 & 0 & 0 & 0 & -1 & 0 & 0\\ 0 & 0 & 0 & 0 & 0 & -1 & 0 & 1 & 0\\ 0 & 0 & 0 & 0 & 0 & 0 & 1 & 0 & -1\\ 0 & 0 & 0 & 0 & -1 & 0 & 0 & -1 & 0 \end{array}\right]$$

It can be easily verified that for the signed graph S as shown in Fig. 2, $C_{E}\left(S\right)$ is S-consistent. There exists only one cycle 2−3−4−5−6−7−8 which is heterogeneous. Given cycle contains two negative sections 2−3−4and5−6−7 each of length 2. Both the negative sections have length > 1 and total negative sections and total edges are of length 2. Thus both the counts are of same parity. Also, for the vertex 2, d(2)≥3, $d^{-}\left(v\right)\;\leq \;1$ and there exists no negative section containing vertex 2. All conditions are satisfied for S, therefore, for the signed graph S, $C_{E}\left(S\right)$ is S-consistent.

### Algorithm to find S-consistency in Common-Edge signed graph

Here, by vertex we mean adjacency matrix of the signed graph S of order n. NbPositiveEdge, NbTotalEdge and NbNegativeEdge denotes count of positive, total and negative edges incident to every node. by NbEdgesInSection, NbNegativeSections, NbNodesInPath and TotalNegativeEdges total edges in each section, total negative section of length >1, total nodes in the path and total negative sections of length >1, respectively, are defined.

Step 1. To obtain $C_{E}\left(S\right)$ refer to [10,11].
Step 2. // Checking a(i.) and a(ii.) condition
Step 3. Repeat Step 3 to 5 for Node i=1 to n
Step 4. Assign path.push-back(i)
 IsNodeInPath[i] = true
Step 5. FindCycle(n, i, &path, IsNodeinPath, IsPathEval uatedforNode, Evaluatepath)
 If return is 0,
 Write As condition is false, $C_{E}\left(S\right)$ is not S-consistent and Go to Step 15 and Step 6. // checking (b) condition
Step 7. Repeat Step 7 to Step 13 for Node i=1 to n
Step 8. Assign NbPositiveEdges = 0
 NbNegativeEdges = 0
 NbtotalEdges = 0
Step 9. Repeat Step 9 to Step 10 for j=1 to n
Step 10. if (vertices [i][j]==1) Set NbPositiveEdges ++ if (vertices [i][j]==−1) NbNegativeEdges++
 if ((vertices[i][j]==−1) ǁ (vertices[i][j]==1)) NbTotalEdges++
Step 11. check if(NbTotalEdges > = 3)
 check if(NbNegativeEdges >2), if yes,
 Write ‘b’ condition is false, hence $C_{E}\left(S\right)$ is not S-consistent and Goto Step 15
Step 12. if(NbTotalEdges > = 3), Set Assign path1.push-back(i) IsNodeInPath1[i] = true
Step 13. FindCycle(n, i, &path1, IsNodeinPath1, IsPath EvaluatedforNode1, Evaluatepath1)
 If it return 0,
 Write ‘b’(ii) is false, thus $C_{E}\left(S\right)$ is not S-consistent and Goto Step 15 Step 14. Write $C_{E}\left(S\right)$ is S-consistent”
Step 15. Exit

### Evaluate(path) function

Step 1. Assign NbNodesInPath = path → size ()
Step 2. Assign StartIndex =–1 and PositiveEdgePresent = false
Step 3. Second condition
 Repeat Step 3 to Step 4 for i = 1 to NbNodesInPath
Step 4. if (vertices [(* path) [i % NbNodesInPath]][(* path)[(i+1)
 % NbNodesInPath]] ==1), Update
 PositiveEdgePresent= true
 StartIndex =i
 break;
Step 5. if (!PositiveEdgePresent)
 Write Cycle is homogenous and cycle length is =”
 NbNodesInPath
 return (NbNodesInPath % 2 ==0)
Step 6. Else Assign NbNegativeSections = 0 NbEdgesInSection = 0
 TotalNegativeEdges = 0
 IsCurrentSectionNegative = false
Step 7. Repeat Step 7 to Step 14 for i = StartIndex to (StartIndex
 + NbNodesInPath + 1)
Step 8. if (vertex[(* path)[i % NbNodesInPath]][(* path)[(i + 1)
 % NbNodesInPath]] ==−1) if (!IsCurrentSectionNegative)
 Write “Started negative section at” (* path) [i % NbNodesInPath]
 and Update IsCurrentSectionNegative = true NbEdgesInSection++
Step 9. else if (IsCurrentSectionNegative)
 Write Negative section finishes at (* path)[i % NbNodesIn Path] “ of size ” NbEdgesInSection
 if (NbEdgesInSection >1), If yes
 Update nbNegativeSections++
 TotalNegativeEdges= TotalNegativeEedges + NbEdgesInSection if (NbEdgesInSection >2) return(false)
 IsCurrentSectionNegative = false
Step 10. Print Negative sections of length >1 NbNegativeSections Print Negative edges in negative sections
 of length >1 = TotalNegativeEdges
Step 11. if((NbNegativeSections % 2 == 0 &&(TotalNegativeEdges
 % 2 == 0)),
 return (true)
Step 12. if((NbNegativeSections %2!=0)&&(TotalNegativeEdges
 %2!=0)),
 return (true)
Step 13. return(false)

Here procedure/function **FindCycle1** is used to find all cycles from adjacency matrix starting with vertex where degree is ≥ 3. This function returns true if size of edges in every section is at most one. If size increases by one, function returns false.

### Function evaluate1 (path1)

Step 1. Assign NbNodesInPath1 = path1 → size()
Step 2. Assign StartIndex = –1 and PositiveEdgePresent = false
Step 3. Repeat Step 3 to Step 4 for i = 1 to NbNodesInPath1
Step 4. if (vertex [(* path1) [i % NbNodesInPath1]][(* path1)[(i+1)
 % NbNodesInPath1]] == 1),
 if true, update
 PositiveEdgePresent = true
 StartIndex = i
 break;
Step 5. if (!PositiveEdgePresent)
 Path length = NbNodesInPath1
 If (NbNodesInPath1 == 1) return(true)
Step 6. Else Assign NbNegativeSections = 0 NbEdgesInSection = 0
 TotalNegativeEdges = 0
 IsCurrentSectionNegative = false
Step 7. Repeat Step 7 to Step 14 for i = StartIndex to (StartIndex
 + NbNodesInPath1 + 1)
Step 8. if (vertex [(* path1)[i % NbNodesInPath1]][(* path1)[(i + 1)
 % NbNodesInPath1]] == –1)
 if (!IsCurrentSectionNegative)
 Write Negative section starts at (* path1)[i % NbNodesInPath1]
 and update IsCurrentSectionNegative = true
 NbEdgesInSection++
Step 9. else if (IsCurrentSectionNegative)
 Write Negative section finishes at (* path) [i % NbNodesIn Path1] “
 of size ” NbEdgesInSection
 if (NbEdgesInSection < = 1), return(true)
 if (NbEdgesInSection >2) return(false) IsCurrentSectionNegative = false
Step 10. return(false)

### Complexity of the algorithm

To obtain $C_{E}\left(S\right)$ [10] in Step 1, complexity involved = $O(n^{3})$).

In Step 3, every vertex is traversed and Step 5 is executed ‘n' times for every node to obtain cycle. EvaluatePath () function is called to obtain all cycles where every node is travelled.

Complexity is $O\left(n\right)\times \;n\;\times \;n\;=\;O\left(n^{3}\right).$

Repeat Step 7 to Step 14 to find count length of each negative section and total negative section of every cycle, therefore,

Complexity $=\;O\left(n\right)\times \;n\;=\;O\left(n^{2}\right)$

To check condition b(ii.) of the Theorem 2.1, nxn matrix is traversed from Step 7 to Step 10 in the main algorithm to count negative, total and positive edges incident to every node.

Complexity $=\;O(n^{2})$

FindCycle1() function is again executed to find all cycles in Step 13,

Complexity =O(n)×n×n=$O(n^{3})$

Total complexity = $O\left(n^{3}\right)+O\left(n^{3}\right)+O\left(n\right)+O\left(n^{2}\right)+O\left(n^{2}\right)+O\left(n^{3}\right)=O\left(n^{3}\right)$.

Hence complexity involved is $O(n^{3})$.

### Correctness of the algorithm

Theorem 2.2 For any signed graph S, the lower bound complexity to count all positive and negative sections in every cycle of given signed graph S is $O(n^{3})$ where, n≥10.

Proof Let ‘n' is number of vertices and ‘e' be number of edges in the signed graph S. The complexity to find all elementary cycles in a given directed/undirected graph [7] is exponential in nature i.e., complexity is of $O(2^{n})$ and for real world networks it is polynomial in nature [8]. Depth First Search (DFS) algorithm is used randomly for a selected vertex in the graph. During DFS, when discovering an adjacent vertex in the graph, each adjacent vertex is visited before to discover a cycle. The edge between the current vertex (i) and the visited vertex (j) is called a back edge (i,j) and stored in an array to be used later for forming the discovered cycles. When DFS is finished, the algorithm will perform a loop on the array that stores the discovered back edges to form the unique cycles. The cycles will be formed out of discovered back edges by adding to the back edge all edges that form a route from vertex (j) to vertex (i) that grows exponentially.

Now, since our aim is to find first all cycles in the given signed graph S and then all positive and negative sections in that cycle. A signed graph has entries 0, 1 and –1 representing no edge, positive edge and negative edge respectively. The algorithm uses DFS technique to find all cycles of the given signed graph with the same procedure as defined in [7,8] but to find all positive and negative sections of different or same length, a new algorithm is developed by Sinha and; Sethi [12,13] where every node is again traversed to count this length. The loop starts with the first vertex and every cycle is calculated along with negative and positive sections resulting in the time complexity of $O(n^{3})$.

Next step is to prove that this complexity is minimum in nature. Let the complexity be $O(n^{3})$. The algorithm is defined for real networks and for large value of ‘n', it is obvious that $n^{3}\leq 2^{n}$ for n≥10. Thus, our algorithm is lower bound which is restricted to number of vertices greater than or equal to 10.

In other way, mathematically let us suppose that complexity of $O(n^{3})$ is not minimum. Big Oh notation has two basic limitations; it contains no consideration of programming efforts and masks (hides) potentially important constants.

Considering these two limitations,

let $2^{n}<cn^{3}$ (adding constants)

Taking log on both sides

nlog(2)<log(c)+3×log(n)

n<log(c)/log(2)+3/(log(2)log(n)

n<a+blog(n) // a and b are again constants

n<O(log(n)) which is a contradiction

Hence our supposition is wrong. Thus, if potential constants are considered, then the algorithm computed is optimal in nature.

### Geometric interpretation of the algorithm

Different adjacency matrices are defined to check the algorithm.

Example 1 Adjacency matrix of S_1_ and S_2_ is:

$$A\left(S_{1}\right)=\left[\begin{array}{c} \;0\\ -1\\ \;0\\ -1 \end{array}\;\begin{array}{c} -1\\ \;0\\ -1\\ \;0 \end{array}\;\begin{array}{c} \;0\\ -1\\ \;0\\ -1 \end{array}\;\begin{array}{c} -1\\ \;0\\ -1\\ \;0 \end{array}\right]A\left(S_{2}\right)=\left[\begin{array}{c} \;0\\ -1\\ \;0\\ \;0\\ -1 \end{array}\;\begin{array}{c} -1\\ \;0\\ -1\\ \;0\\ \;0 \end{array}\;\begin{array}{c} \;0\\ -1\\ \;0\\ -1\\ \;0 \end{array}\;\begin{array}{c} \;0\\ \;0\\ -1\\ \;0\\ -1 \end{array}\;\begin{array}{c} -1\\ \;0\\ \;0\\ -1\\ \;0 \end{array}\right]$$

Apply algorithm to obtain $C_{E}\left(S\right)$ [10] as defined in Step 1.

The Adjacency matrix of $C_{E}\left(S_{1}\right)$ and $C_{E}\left(S_{2}\right)$ are:

$$A\left(C_{E}(S_{1}\right))=\left[\begin{array}{c} \;0\\ -1\\ \;0\\ -1 \end{array}\;\begin{array}{c} -1\\ \;0\\ -1\\ \;0 \end{array}\;\begin{array}{c} \;0\\ -1\\ \;0\\ -1 \end{array}\;\begin{array}{c} -1\\ \;0\\ -1\\ \;0 \end{array}\right]A\left(C_{E}\left(S_{2}\right)\right)=\left[\begin{array}{c} 0\\ -1\\ \;0\\ -1\\ \;0 \end{array}\;\begin{array}{c} -1\\ \;0\\ -1\\ \;0\\ \;0 \end{array}\;\begin{array}{c} \;0\\ -1\\ \;0\\ \;0\\ -1 \end{array}\;\begin{array}{c} -1\\ \;0\\ \;0\\ \;0\\ -1 \end{array}\;\begin{array}{c} \;0\\ \;0\\ -1\\ -1\\ \;0 \end{array}\right]$$

Firstly, for checking condition a(i) of Theorem 2.1, i.e., first obtain all existing cycles in S. To detect homogenous cycle calculate path length of each cycle and all negative edges in this path. Secondly, if this count is even, then cycle is all negative and of even length and condition a(i) of Theorem 2.1 is satisfied and we have a signed graph which is S-consistent.

In Step 3 to 5, FindCycle () function is used to obtain all the cycle in the signed graph. This function outputs all the paths of the cycle. Evaluate() whether cycle is heterogeneous or homogenous. There does not exist any positive edge in signed graph S_1_ and S_2_, thus both the signed graphs are homogenous. NbNodesInPath is calculated which in signed graph S_1_is 4 (even) and for S_2_ it is 5(odd). Thus, signed graph S_1_ is S-consistent whereas S_2_ is not and hence program terminates after Step 5 in $O(n^{3})$ steps.

Example 2 The adjacency matrices of S_3_ and S_4_ are:

**Figure:**
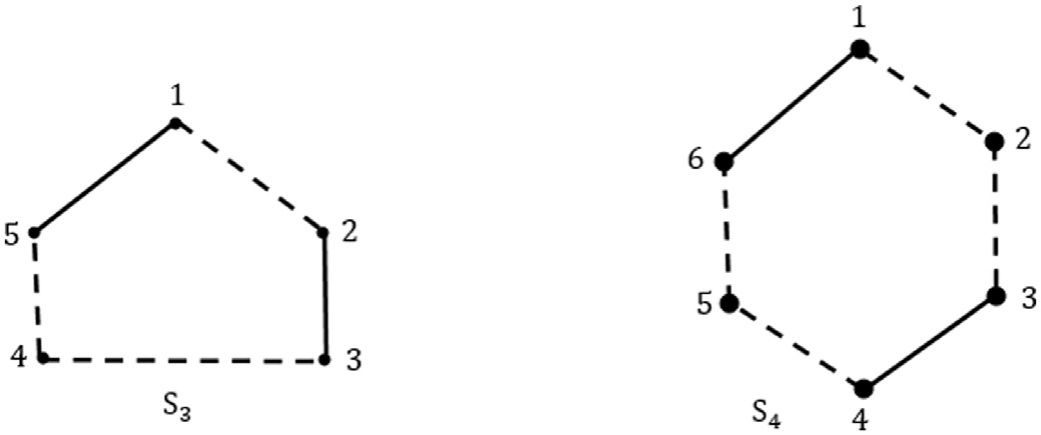

$$A\left(S_{3}\right)=\left[\begin{array}{c} 0\\ -1\\ \;0\\ \;0\\ \;1 \end{array}\;\begin{array}{c} -1\\ \;0\\ \;1\\ \;0\\ \;0 \end{array}\;\begin{array}{c} \;0\\ \;1\\ \;0\\ -1\\ \;0 \end{array}\;\begin{array}{c} \;0\\ \;0\\ -1\\ \;0\\ -1 \end{array}\;\begin{array}{c} \;1\\ \;0\\ \;0\\ -1\\ \;0 \end{array}\right]A\left(S_{4}\right)=\left[\begin{array}{c} 0\\ -1\\ \;0\\ \;0\\ \;0\\ \;1 \end{array}\;\begin{array}{c} -1\\ \;0\\ -1\\ \;0\\ \;0\\ \;0 \end{array}\;\begin{array}{c} \;0\\ -1\\ \;0\\ \;1\\ \;0\\ \;0 \end{array}\;\begin{array}{c} \;0\\ \;0\\ \;1\\ \;0\\ -1\\ \;0 \end{array}\;\begin{array}{c} \;0\\ \;0\\ \;0\\ -1\\ \;0\\ -1 \end{array}\;\begin{array}{c} \;1\\ \;0\\ \;0\\ \;0\\ -1\\ \;0 \end{array}\right]$$

To check condition a(ii) of Theorem 2.1, first obtain a cycle. Check if any positive edge exists in the cycle. Cycle is heterogeneous if positive edge exists. Negative edges (entries) in the cycle are calculated. If positive edge is preceded with only one negative edge in the path of cycle, negative section exists and updates edges in NbEdgesInSection. If the count >1, then increase NbNegativeSections by 1 and TotalNegativeedges by NbEdgesInSection. If NbNegativeEdgesInSection exceeds 1, then stop the process and search for new cycle. This way total negative sections of length >1 and total negative edges in the negative sections of length >1 are found. Both NbNegativeSections and TotalNegativeEdges must be of same parity. If both the counts are odd or even then go to next condition else say that condition is false and hence $C_{E}\left(S\right)$ is not S-consistent.

Signed graph *S*_3_ contains two negative sections, 3−4−5 of length 2 and 1−2 of length 1. Now count negative sections where length >1, i.e NbNegativeSections = 1. Also, total negative edges where length of the negative section >1, TotalNegativeEdges = 2. Since both are of different parity, S_3_ does not satisfy the given condition and program is terminated after Step 12 of Evaluate () function again in $O(n^{3})$ steps.

Similarly, S_4_ contains two negative sections, 4−5−6 and 1−2−3 both of length 2. Here total negative sections of length >1 i.e., NbNegativeSections = 2 and total negative edges in above calculated negative sections i.e, TotalNegativeEdges = 4. Since, both are of same parity i.e even, therefore, S_4_ holds true for a(ii) and check next condition. Degree of each vertex is calculated and no vertex exists with degree > 3, thus, program again terminates in $O(n^{3})$ steps.

## Conclusion and future scope

An algorithmic characterization is defined for a given S whose $C_{E}\left(S\right)$ is S-consistent in $O(n^{3})$ steps. An adjacency matrix is taken as data with values as 1, –1 and 0. Network of ‘n' vertices can be restricted depending on number of connections as it affects the time complexity. We can represent any matrix as an image and also vice-versa. Encryption and decryption mechanism can be applied to an image through matrices. By using the algorithms defined to obtain Common-Edge signed graph [10] from a given S and Common-Edge root signed graph of S see [10,11], decryption and encryption algorithm can be applied to an image where $C_{E}\left(S\right)$ is S-consistent. Such application has already been discussed by the authors for another derived network Line signed graphs see [12]. The same can be applied to $C_{E}\left(S\right)$ too.

## Declaration of Competing Interest

The authors declare that they have no known competing financial interests or personal relationships that could have appeared to influence the work reported in this paper.

## Data Availability

No data was used for the research described in the article.
